# Patterns of organizing pneumonia and microinfarcts as surrogate for endothelial disruption and microangiopathic thromboembolic events in patients with coronavirus disease 2019

**DOI:** 10.1371/journal.pone.0240078

**Published:** 2020-10-05

**Authors:** Katharina Martini, Christian Blüthgen, Joan Elias Walter, Thi Dan Linh Nguyen-Kim, Friedrich Thienemann, Thomas Frauenfelder

**Affiliations:** 1 Institute of Diagnostic and Interventional Radiology, University Hospital Zurich, Zurich, Switzerland; 2 Faculty of Medicine, University of Zurich, Zurich, Switzerland; 3 Department of Internal Medicine, University Hospital Zurich, Zurich, Switzerland; University Magna Graecia of Catanzaro, ITALY

## Abstract

**Background:**

To evaluate chest-computed-tomography (CT) scans in coronavirus-disease-2019 (COVID-19) patients for signs of organizing pneumonia (OP) and microinfarction as surrogate for microscopic thromboembolic events.

**Methods:**

Real-time polymerase-chain-reaction (RT-PCR)-confirmed COVID-19 patients undergoing chest-CT (non-enhanced, enhanced, pulmonary-angiography [CT-PA]) from March-April 2020 were retrospectively included (COVID-19-cohort). As control-groups served 175 patients from 2020 (cohort-2020) and 157 patients from 2019 (cohort-2019) undergoing CT-PA for pulmonary embolism (PE) during the respective time frame at our institution. Two independent readers assessed for presence and location of PE in all three cohorts. In COVID-19 patients additionally parenchymal changes typical of COVID-19 pneumonia, infarct pneumonia and OP were assessed. Inter-reader agreement and prevalence of PE in different cohorts were calculated.

**Results:**

From 68 COVID-19 patients (42 female [61.8%], median age 59 years [range 32–89]) undergoing chest-CT 38 obtained CT-PA. Inter-reader-agreement was good (k = 0.781). On CT-PA, 13.2% of COVID-19 patients presented with PE whereas in the control-groups prevalence of PE was 9.1% and 8.9%, respectively (p = 0.452). Up to 50% of COVID-19 patients showed changes typical for OP. 21.1% of COVID-19 patients suspected with PE showed subpleural wedge-shaped consolidation resembling infarct pneumonia, while only 13.2% showed visible filling defects of the pulmonary artery branches on CT-PA.

**Conclusion:**

Despite the reported hypercoagulability in critically ill patients with COVID-19, we did not encounter higher prevalence of PE in our patient cohort compared to the control cohorts. However, patients with suspected PE showed a higher prevalence of lung changes, resembling patterns of infarct pneumonia or OP and CT-signs of pulmonary-artery hypertension.

## Introduction

Recent observations suggest that respiratory failure in novel coronavirus disease 2019 (COVID-19) is not driven by the development of acute respiratory distress syndrome alone, but that microvascular thrombotic processes may play a role [1]. This may have important consequences for the diagnostic and therapeutic management of these patients. There is a strong association between D-dimer levels, disease progression and chest CT features suggesting venous thrombosis. In addition, various studies in patients with COVID-19 have shown a very strong association between increased D-dimer levels and severe disease/poor prognosis [1].

Different studies have shown that COVID-19 is characterized by hypercoagulability and endothelial dysfunction [2,3]. This, together with the commonly observed higher occurrence of altered coagulopathy in sepsis [4], makes COVID-19 patients prone to thromboembolic events. In fact, some trials demonstrated higher prevalence of cardiac, cerebrovascular, and pulmonary vascular events in COVID-19 patients [2,5–7]. However, since detection of typical lung imaging features of COVID-19 does not require intravenous contrast agent, patients with COVID-19 pneumonia are generally imaged with non-contrast chest CT. One hypothesis is, that the high mortality observed among COVID-19 patients may be partly due to undiagnosed pulmonary embolism (PE) and pulmonary *in situ* thrombosis, outlining the possible role of CT pulmonary angiography (CT-PA) in patients with rapid clinical worsening [2,8] for appropriate management.

Micro-embolism caused by microscopic endothelial disruption, however, are too small to be captured on CT. The only surrogate for the underlying pathophysiologic process might be secondary lung changes such as peripheral wedge-shaped consolidation. The pathophysiology might be comparable to that of organizing pneumonia (OP), where alveolar epithelial injury leads to an activation of the coagulation cascade and fibrin deposition within the alveoli causing the typical parenchymal changes on high resolution CT (HRCT) of the lungs [9].

To date, studies showed a significantly higher percentage of macroscopic PE in COVID-19 patients [2,5]. However, the dark figure of unreported microscopic thromboembolic events in the lungs remain unclear. Therefore, the purpose of the study was to investigate possible typical parenchymal lung changes resembling patterns of infarct pneumonia or OP as surrogate for microscopic thromboembolic events in COVID-19 patients.

## Materials and methods

### Patient population

In this retrospective cohort study, we included data of consecutively admitted adults with COVID-19 to the isolation wards and intensive care units at the University Hospital Zurich, one of the largest tertiary care centres in Switzerland. COVID-19 was confirmed in all cases using real-time polymerase chain reaction (RT-PCR) for severe acute respiratory syndrome virus 2 (SARS-CoV-2). Clinical information and diagnostic results were extracted from our hospital electronic medical records. The swiss ethics committee (*swissethics*, section Zurich) approved the study and written informed consent was waived due to the retrospective nature of the study.

Patients undergoing CT-PA for suspected PE at our institution from March to April 2020 (cohort 2020 –tested negative for COVID-19 infection) and March to April 2019 (cohort 2019 –no test available; but acquired before the outbreak of COVID-19) in the same time frame served as two control cohorts.

### Eligibility criteria to perform non-contrasted CT chest and CT-PA

Chest X-ray was performed in all patients with COVID-19 as baseline investigation on admission. In case of a normal chest X-ray and clinical suspected pneumonia, a CT chest without contrast was performed. CT-PA was performed if at least one of the following criteria was present: 1) clinical signs and symptoms of deep vein thrombosis, 2) tachypnoea, 3) decreased oxygen saturation, or 4) high oxygen demand.

### CT protocol

Single-energy CT was performed in all patients on third-generation CT scanner (SOMATOM Force, SOMATOM Definition AS, or SOMATOM Definition Flash; Siemens Healthcare, Forchheim, Germany) equipped with an integrated high-resolution detector (Stellar Technology; Siemens). Scanning parameters were as follows: CT was performed at 100 kVp with quality reference current-time product of 80 mAs, a pitch of 1.2, gantry rotation time 0.5 s, slice acquisition of 192 x 0.6 mm by means of a z-flying focal spot. The onsite CT technician explained the breathing instructions to the patient.

For CT-PA a double-syringe power injector (CT Exprés, Bracco—former Swiss Medical Care, Switzerland) infused IV contrast via an antecubital, subclavian or internal jugular venous access. 80 mL of IV contrast (Iopromide, Ultravist, 300 mg J/ml, Bayer HealthCare, Germany) was followed by 50mL saline bolus, both at a flow rate of 4 mL/s. Bolus tracking was performed with a threshold at 100 HU (at 100 kVp) in the main pulmonary artery, with a trigger delay of 10 seconds.

All images were reconstructed with advanced modelled iterative reconstruction (ADMIRE, Siemens Healthcare, Forchheim, Germany) at a strength level of 3, using a slice thickness of 1.5 mm, an increment of 1 mm, and a tissue convolution kernel (Bl34). The image matrix was 512 x 512 pixels.

### Image analysis

The images were presented to two independent readers (TF and TDLNK, both attending radiologists with 20 and 11 years of experience and blinded to clinical information.

#### Evaluation of LE specific findings

Both readers independently assessed the images for the presence and location (central vs. segmental vs. sub-segmental) of PE and presence of peripheral wedge-shaped consolidation as signs of infarct pneumonia. Furthermore, readers measured the right/left ventricular ratio (RV/LV ratio), the width of the pulmonary artery and the ratio between pulmonary artery and aorta (P/A ratio) in order to capture indirect signs for raised pulmonary arterial pressure and right heart failure. According to Ende-Verhaar et al. [10] RV/LV ratio was calculated by dividing the maximal distance between the ventricular endocardium and the interventricular septum, perpendicular to the long axis of the heart measured on standard axial views. The maximum dimensions for both ventricles were used for measurements.

**Fig 1:**
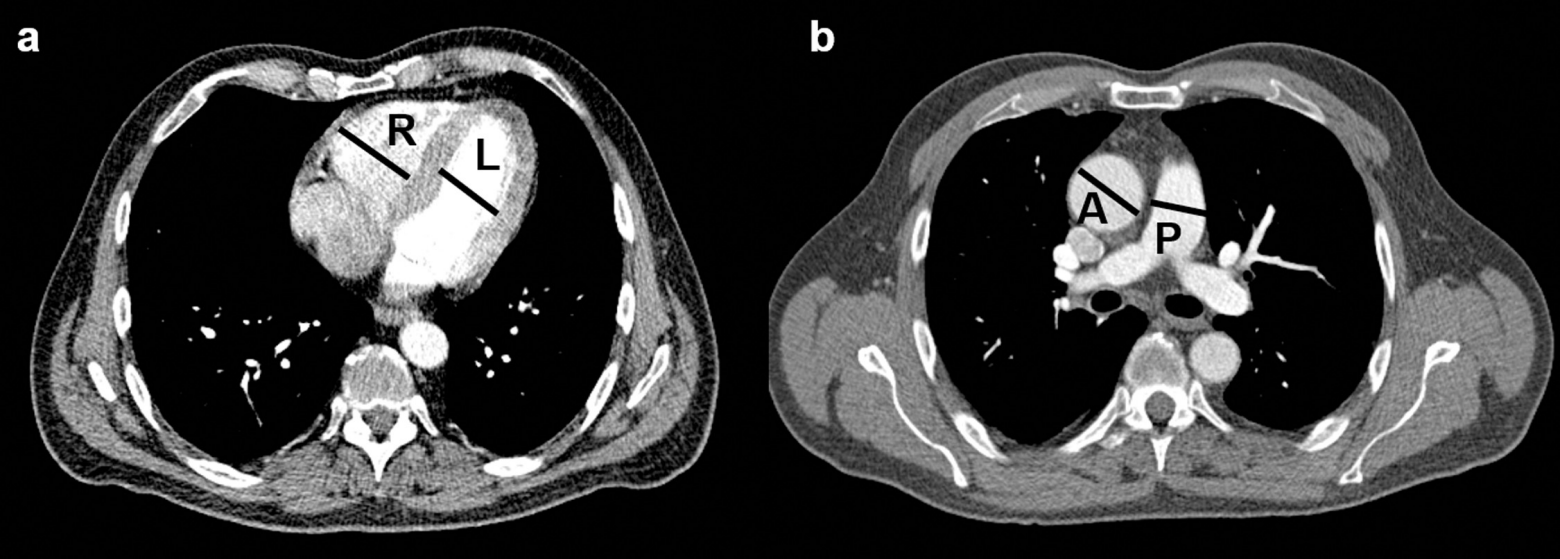
CT measurements. a) Right/left ventricular ratio was calculated by dividing the maximal distance between the ventricular endocardium and the interventricular septum, perpendicular to the long axis of the heart measured on standard axial views. The maximum dimensions for both ventricles where used for measurements. b) The pulmonary artery and ascending aorta were assessed on a transverse image at the level of the pulmonary artery bifurcation. Vessel diameters were obtained by measuring the widest diameter vertical to the long axis of the main pulmonary artery.

According to Lee et al. [11] the pulmonary artery and ascending aorta were assessed on a transverse image at the level of the pulmonary artery bifurcation. Vessel diameters were obtained by measuring the widest diameter vertical to the long axis of the main pulmonary artery (Fig 1).

While PA diameter and P/A ratio was performed in the entire datasets, RV/LV ratio could only be measured in contrast-enhanced CT scans.

#### Evaluation of lung parenchymal changes

Further parameters for COVID-19 pneumonia and OP were assessed (for details see Table 1).

**Table 1:** Imaging findings in the COVID-19-cohort–Lung parenchyma changes. Computed tomography pulmonary angiography (CT-PA), Number of cases (n), Predominant (Pred.), Ground-glass opacification (GGO), Cryptogenic organizing pneumonia (COP), bronchocentric pattern: conspicuous cuffs around larger bundles with extension to lung periphery, band like pattern: thick radial bands (≥8mm width), sometime containing a small air bronchogram, which distinguishes it from linear atelectasis, crazy paving: GGO and superimposed interlobular septal thickening, pulmonary artery (PA), diameter (dia.), pulmonary artery aorta ratio (P/A ratio), right/left ventricular ratio (RV/LV ratio).

Imaging findings–Lung parenchyma changes					
	Total (n = 68)	Non CT-PA group (n = 30)	CT-PA group (n = 38)	p-value	Kappa
***Main pattern***					
*GGO*, *n (%)*	39 (57.4)	14 (46.7)	25 (65.8)	0.580	0.768
*Consolidation*, *n (%)*	28 (41.2)	15 (50.0)	13 (34.2)		
*Equal*, *n (%)*	1 (1.5)	1 (3.3)	0 (0)		
***Distribution***					
*Unilateral*, *n (%)*	4 (5.9)	1 (3.3)	3 (4.4)	0.716	0.259
*Pred*. *upper lobes*, *n (%)*	1 (1.5)	0 (0)	1 (2.6)	0.433	0.938
*Pred*. *lower lobes*, *n (%)*	34 (50.0)	16 (53.3)	18 (47.4)		
*Diffuse*, *n (%)*	33 (48.4)	14 (46.7)	19 (50.0)		
*Pred*. *central*, *n (%)*	1 (1.5)	0 (0)	1 (2.6)	0.099	0.686
*Pred*. *peripheral*, *n (%)*	40 (58.5)	21 (70.0)	19 (50.0)		
*Diffuse*, *n (%)*	27 (39.7)	9 (30.0)	18 (47.4)		
*Amount (<20%; 20–50%; >50%)*	10 / 29 / 29	5 / 18 / 7	5 / 11 / 22	0.085	0.809
***GGO***					
*present*, *n (%)*	60 (88.2)	26 (86.6)	32 (47.1)	0.776	0.782
*Single* : *multiple*	4 : 56	2 : 28	2 : 30	0.947	0.770
*Only central*, *n (%)*	2 (2.9)	0 (0)	2 (5.3)	0.390	0.824
*Only peripheral*, *n (%)*	34 (50)	14 (46.7)	20 (52.6)		
*Diffuse*, *n (%)*	28 (41.2)	16 (53.3)	12 (31.6)		
***Consolidation***					
*present*, *n (%)*	55 (80.9)	25 (83.3)	30 (78.9)	0.698	0.946
*With air-bronchogram*, *n (%)*	40 (58.9)	15 (50.0)	25 (65.8)	0.210	0.866
*Single* : *multiple*	7 : 48	6 : 19	1 : 29	0.431	0.789
*Only central*, *n (%)*	0 (0)	0 (0)	0 (0)	0.727	0.816
*Only peripheral*, *n (%)*	45 (66.2)	23 (76.7)	22 (57.9)		
*Diffuse*, *n (%)*	11 (16.2)	2 (6.7)	9 (23.7)		
***Additional findings***					
*Septal thickening*, *n (%)*	16 (23.5)	8 (26.7)	8 (21.1)	0.331	0.748
*Fine reticular opacity*, *n (%)*	29 (42.6)	10 (33.3)	19 (50.0)		
*Micronodules (≤ 4mm)*, *n (%)*	5 (7.4)	3 (10)	2 (5.3)	0.670	0.790
*Lager nodules (up to 10mm*, *n (%)*	3 (4.4)	1 (3.3)	2 (5.3)		
*Vascular thickening*, *n (%)*	1 (1.5)	0 (0)	1 (2.6)	0.393	1.000
*Bronchial thickening*, *n (%)*	1 (1.5)	0 (0)	1 (2.6)	0.393	0.139
*Air bronchogram*, *n (%)*	40 (58.8)	14 (46.7)	26 (68.4)	0.136	0.964
*Halo sign*, *n (%)*	5 (7.4)	3 (10.0)	2 (5.3)	0.405	0.731
*Pleural thickening*, *n (%)*	6 (8.8)	4 (13.3)	2 (5.3)	0.206	0.638
*Pleural effusion*, *n (%)*	8 (11.8)	4 (13.3)	4 (10.5)	0.635	1.000
*Lymphadenopathy*, *n (%)*	17 (25.0)	7 (23.3)	10 (26.3)	0.922	0.956
***Specific changes for PE and right heart failure***					
*Median PA dia*. *[cm] (range)*	30 (19–38)	29 (23–38)	30 (19–38)	0.776	0.876
*Median P/A ratio (range)*	0.91 (0.6–1.2)	0.88 (0.6–1.2)	0.93 (0.7–1.2)	**0.022**	0.911
*Median RV/LV ratio (range)*	0.93 (0.7–1.5)*	0.88 (0.7–1.1)*	0.92 (0.7–1.5)	0.603	0.964
*Triangular shaped peripheral GGO*, *n (%)*	12 (17.6)	4 (13.3)	8 (21.1)	0.446	0.465
***COP specific changes***					
*Rhomboid shape*, *n (%)*	26 (28.2)	10 (33.3)	16 (42.1)	0.426	0.963
*Bronchocentric pattern*, *n (%)*	34 (50.0)	15 (50.0)	19 (50.0)	0.622	0.964
*Band like pattern*, *n (%)*	25 (36.8)	12 (40.0)	13 (34.2)	0.650	0.822
*Reversed halo sign*, *n (%)*	6 (8.8)	4 (13.3)	2 (5.3)	0.206	0.383
*Crazy paving*, *n (%)*	25 (36.8)	7 (23.3)	19 (50.0)	0.068	0.665

* value could only be measured in contras- enhanced CT.

Images were assessed at a random order over a time period of one week. Readers were allowed to modify the window width and level after the initial presentation with a mediastinal and lung window. We chose to not use one of the several proposed scores for COVID-19, since we wanted to focus on specific parenchymal changes, which might get lost when an overall scoring system is used.

Analyses were performed using the picture archiving and communication system (PACS) of our hospital (Impax, Version 6.5.5.1033; Agfa-Gevaert, Mortsel, Belgium) on a high-definition liquid crystal display monitor (BARCO; Medical Imaging Systems, Kortrijk, Belgium).

### Statistical analysis

Statistical analyses were conducted using commercially available software (SPSS, release 26.0; SPSS, Chicago, IL, USA). Continuous variables were expressed as mean +/- standard deviation (SD) while categorical variables were expressed as frequencies or percentages.

Cohen’s Kappa (κ) was used to assess inter-reader agreement for presence of PE. Κ-results were stratified qualitatively by score (slight agreement 0.01–0.20; fair agreement 0.21–0.40; moderate agreement 0.41–0.60; good agreement 0.61–0.80; excellent agreement 0.81–0.99 [12]. Prevalence of PE was calculated for each cohort. Man-Whitney-U-test and a two-sided T-test for unpaired variables was used to test for statistical significance. A two-sided p–value below 0.05 was defined to indicate statistical significance.

## Results

### Patient population

#### COVID-19 patients

**Fig 2:**
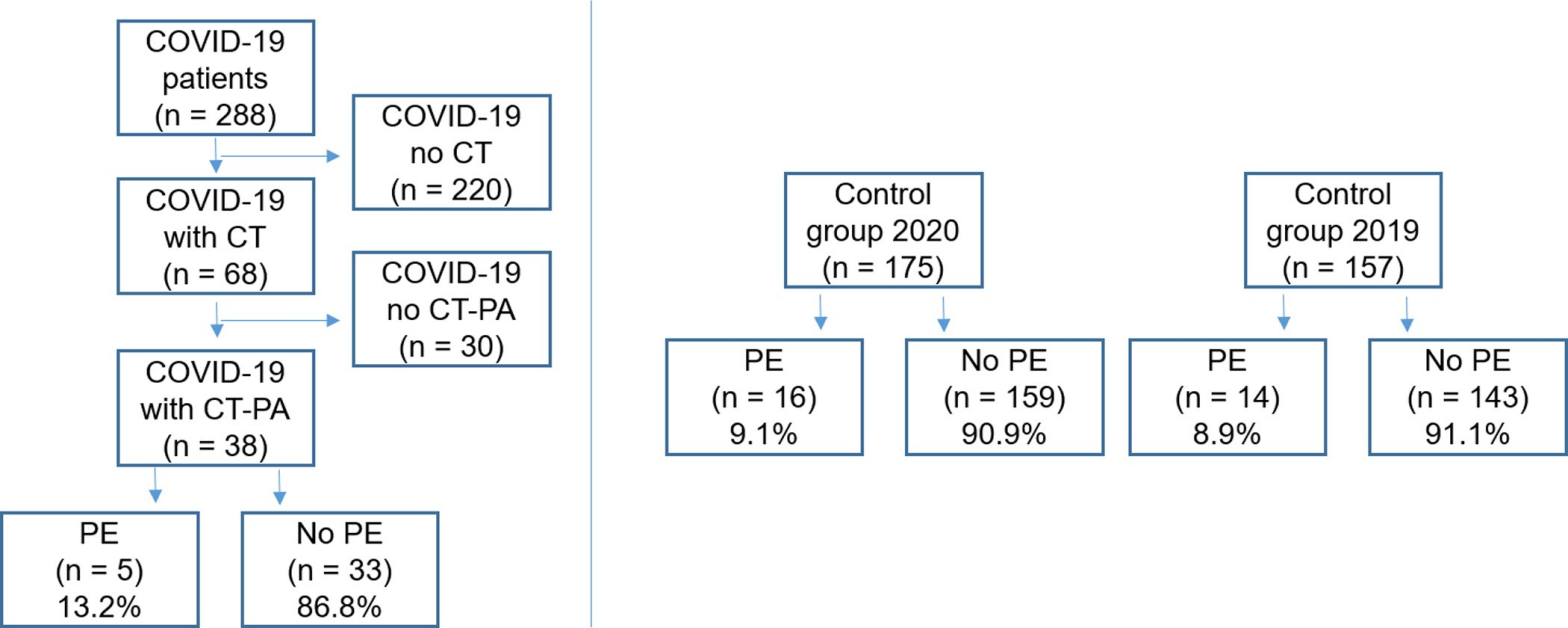
Patient inclusion and prevalence of pulmonary embolism. Pulmonary embolism (PE), number of patients (n), CT pulmonary angiography (CT-PA).

From February to April 2020, sixty-eight patients with confirmed COVID-19 (42 females [61.8%]; median age 59 years [range 32–89]) underwent CT scanning at our hospital and were included in the study. Of these, 38 patients fulfilled the criteria for suspected PE and underwent CT-PA scanning (Table 2 and Fig 2).

**Table 2:** Patient characteristics. Number of patients (n), Years (y), Pulmonary embolism (PE).

Patient characteristics undergoing CTPA				
	COVID-19-cohort (n = 38)	2020-cohort (Non-COVID) (n = 175)	2019-cohort (Non-COVID) (n = 157)	p-value
*Female*, *n (%)*	20 (52.6)	75 (42.9)	68 (43.3)	0.175
*Median age (y)*, *(range)*	59 (32–89)	62 (18–96)	62 (17–94)	0.989
*Number of patients with PE (n)*, *(%)*	5 (13.2%)	16 (9.1%)	14 (8.9%)	0.452

### Non-COVID-19 patients (cohort 2019 and cohort 2020)

Cohort 2019: In the respective time frame from March to April 2019, 157 consecutive patients (89 females [56.7%]; median age 62 years [range 17–94]) who were referred to our hospital with suspected pulmonary embolism were retrieved and included.

Cohort 2020: In the time frame from March to April 2020 175 consecutive patients (75 females [42.9%]; median age 62 years [range 18–96]) who were referred to our hospital with suspected pulmonary embolism were retrieved and included.

### Clinical findings

Mean time from onset of clinical symptoms was of 7.2 days (SD ±8.9) on date of CT. The following comorbidities were present: cardiovascular disease (26.5%), arterial hypertension (51.5%), diabetes (35.3%), chronic renal failure (26.5%), and chronic pulmonary disease (19.1%).

23.5% of patients (n = 16) were receiving anticoagulative therapy at time point of CT, which mostly was performed already on admission. Mean value of D-dimer was of 2.7 mg/l (SD±2.7) (non-CT-PA group 1.8 mg/l ±1.2 vs. CT-PA group 3.1 mg/l ±3.1; p = 0.269). Detailed information on clinical findings can be found in **Table 3**.

**Table 3:** Clinical findings in COVID-19-cohort. Computed tomography pulmonary angiography (CT-PA), Number of patients (n), Acute respiratory distress syndrome (ARDS), Intensive care unit (ICU), International normalized ratio (INR).

Clinical findings				
	Total (n = 68)	Non-CT-PA group (n = 30)	CT-PA group (n = 38)	p-value
***Mean Time since onset of symptoms (days)***	7.2±8.9	5.8±7.9	8.3±9.4	0.246
***Body mass index***				
*≤25 kg/m*^*2*^, *n (%)*	22 (32.4)	12 (40.0)	8 (21.1)	0.089
*<25–30 kg/m*^*2*^, *n (%)*	24 (35.3)	8 (26.7)	16 (42.1)	0.186
*>30 kg/m*^*2*^, *n (%)*	22 (32.4)	8 (26.7)	14 (36.8)	0.373
***Coagulation***				
*D-Dimer at time of CT*	2.7±2.7	1.8±1.2	3.1±3.1	0.269
*Thrombocytes /μl*	234.8±123.3	224.5±138.9	243.5±109.7	0.531
*Quick*	83.3±22.9	83.8±24.6	83.9±22.4	0.987
*INR*	1.2±0.4	1.2±0.3	1.2±0.4	0.730
*Prothrombin time (s)*	13.4±10.5	14.8±4.8	12.3±4.0	0.349
*Anticoagulative therapy*, *n (%)*	16 (23.5)	8 (26.7)	8 (21.1)	0.083
***Comorbidities***				
*Cardiovascular disease*, *n (%)*	18 (26.5)	8 (26.7)	10 (26.3)	0.974
*Arterial hypertension*, *n (%)*	35 (51.5)	17 (56.7)	18 (47.4)	0.446
*Diabetes mellitus*, *n (%)*	24 (35.3)	9 (30.0)	15 (39.5)	0.417
*Chronic kidney disease*, *n (%)*	18 (26.5)	9 (30.0)	9 (23.7)	0.558
*Chronic pulmonary disease*, *n (%)*	13 (19.1)	7 (23.3)	6 (15.8)	0.432
*Hepatitis or Liver cirrhosis*	6 (8.8)	2 (6.7)	4 (10.5)	0.577
*Malignancy*	12 (17.6)	8 (26.7)	4 (10.5)	
***ARDS***, *n (%)*	29 (42.6)	11 (36.7)	18 (47.4)	0.376
***Treatment type at diagnosis***				
*Out of hospital*, *n (%)*	6 (8.8)	1 (3.3)	5 (13.2)	0.156
*In hospital*, *n (%)*	46 (67.6)	21 (70.0)	25 (65.8)	0.712
*ICU no mechanic ventilation*, *n (%)*	6 (8.8)	4 (13.3)	2 (5.3)	0.244
*ICU mechanic ventilation*, *n (%)*	10 (14.7)	4 (13.3)	6 (15.8)	0.776

### Imaging findings

Mean inter-reader agreement for imaging findings was good (k = 0.781).

#### COVID specific findings

Predominant findings in COVID-19 patients where ground glass opacities (GGO, 57.4%) with a basal, subpleural distribution, followed by consolidations (41.2%) with a similar distribution pattern (Table 1). The majority of patients showed bilateral lung changes (94.1% vs 6.9%; respectively).

Additional findings such as lymphadenopathy (defined as enlarged lymph nodes > 1cm short axis) or pleural effusion were relatively rare (11.8% and 25.0%, respectively).

#### PE specific findings

**Fig 3:**
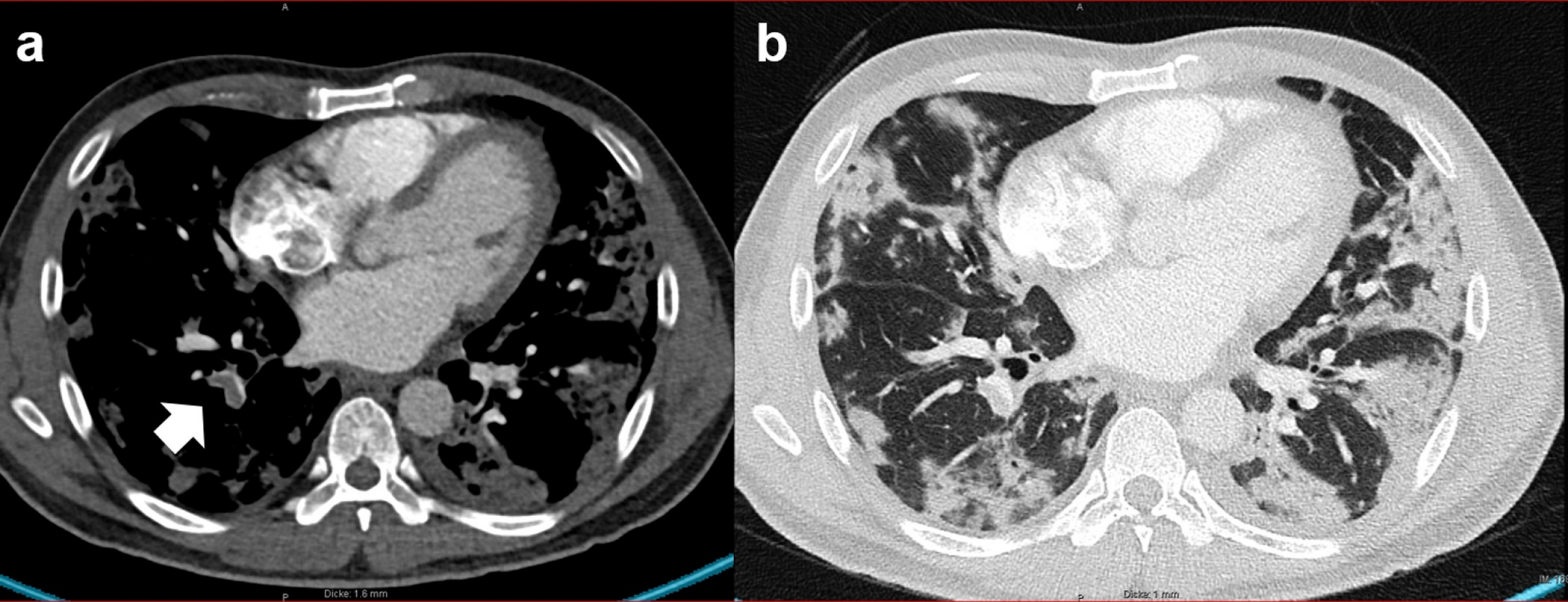
Pulmonary embolism and infarct pneumonia. 34-year-old male patient with COVID-19 pneumonia and a) segmental pulmonary embolism (arrow) in the branch of the lower lobe artery. b) shows wedge shape subpleural consolidation resembling the appearance of infarct pneumonia.

In the COVID-19 cohort, five patients presented with PE (13.2%), while in cohort 2019 14 patients (8.9%) and cohort 2020 16 patients (9.1%) were diagnosed with PE (p = 452) (Table 2 and Figs 1 and 3).

COVID-19 patients suspected with PE tended to show more triangular or wedge shaped peripheral GGO compared to COVID-19 patients without suspected PE (21.1% vs. 13.3% respectively, p = 0.465).

Half of COVID-19 patients with patterns resembling infarct pneumonia on CT showed signs of right heart failure and pulmonary hypertension on CT (increased diameter of the pulmonary artery, lowered RV/LV ratio or increase in P/A ratio). Patients who underwent CT-PA and patients, where CT showed peripherally wedge-shaped consolidation resembling patterns of infarct pneumonia had significantly higher P/A ratio compared to their counterparts (P/A ratio 0.98 vs 0.91, p = 0.022 and P/A ratio 0.93 vs 0.88, p = 0.024; respectively). Median RV/LV ratio tended to be higher in patients showed CT sign of infarct pneumonia compared to patients without this pattern (RV/LV 0.88 vs 0.94, respectively; p = 0.289 (Tables 1 and 4).

**Table 4:** Imaging findings in the COVID-19-cohort–CT signs for right heart failure/pulmonary artery hypertension. Peripheral wedge-shaped consolidation (PWSC) as imaging biomarker for microinfarction, number of cases (n), pulmonary artery (PA), diameter (dia.), pulmonary artery aorta ratio (P/A ratio), right/left ventricular ratio (RV/LV ratio).

	Total (n = 68)	PWSC (n = 12)	No PWSC (n = 56)	p-value
***CT signs for right heart failure / PA hypertension***				
*Median PA dia*. *[cm] (range)*	30 (19–38)	29 (19–38)	31 (26–38)	0.175
*Median P/A ratio (range)*	0.91 (0.6–1.2)	0.91 (0.6–1.2)	0.98 (0.8–1.1)	**0.024**
*P/A ratio > 1*, *n (%)*	18 (26.5)	6 (50.0)	12 (21.4)	**0.042**
*RV/LV ratio > 1*, n (%)*	12 (24.5)	4 (40.0)	8 (20.5)	0.201
Median RV/LV ratio (range)*	0.93 (0.7–1.5)	0.88 (0.7–1.5)	0.94 (0.8–1.3)	0.289

* value could only be measured in contras- enhanced CT.

#### OP specific findings

**Fig 4:**
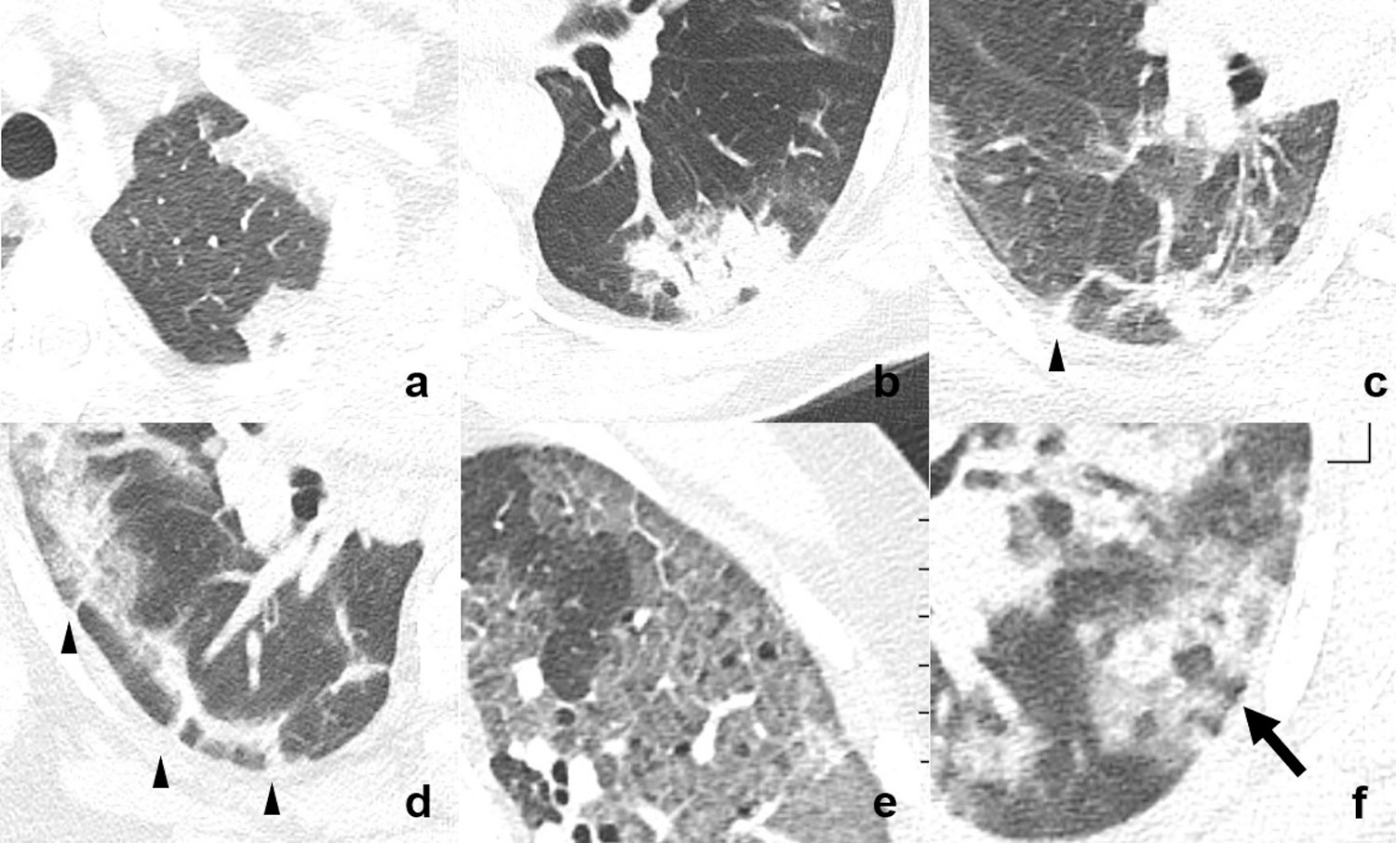
Typical signs of organizing pneumonia. a) Rhomboid shape, b) bronchocentric pattern meaning conspicuous cuffs around larger bronchovascular bundles, c-d) band like pattern meaning thick radial bands curved toward non-thickened pleura (arrow-head), e) crazy paving meaning GGO with superimposed interlobular septal thickening, and f) nodular reversed halo sign or atoll pattern (arrow).

COVID-19 patients showed parenchymal changes typical of OP, namely rhomboid shape (18.2%), bronchocentric pattern (50.0%), band like pattern (36.8%), and crazy paving (36.8%). The nodular reversed halo sign was only seen in 8.8% of COVID-19 patients (Fig 4).

## Discussion/Conclusion

Hypercoagulability is present in severe infection [13–15] and has also been reported in COVID-19: Recent studies showed, that COVID-19 patients have increased D-dimers, fibrin degradation product (FDP), and fibrinogen levels compared to healthy volunteers [16]. These alterations in the state of coagulation reportedly lead to a higher incidence of thromboembolic events [2].

In contrary to this, we could not show a higher prevalence of PE in our COVID-19 cohort compared to control groups from 2019 and 2020. However, we encountered lung parenchyma changes, mimicking those of infarct pneumonia and OP.

Normally, the annual incidence of venous thromboembolism is reported to be between 20–70 cases per 100.000 [17,18]. One third of those patients have acute PE, while two thirds remain isolated deep vein thrombosis [19]. The current approach to patients with suspected PE is based on a clinical adjudication of patients into a high (>15%) and a non-high risk group of early PE-related death and CT-PA is the first-choice imaging modality in the former [17].

A hypothesis is, that the high mortality observed among COVID-19 patients may be partly due to unrecognized PE and pulmonary *in situ* thrombosis. However, current guidelines advocate the use of non-contrast chest CT for the diagnosis, severity assessment, and monitoring of COVID-19 [20]. Although the mortality of COVID-19 infection seems relatively low, patients with severe or critical disease are at high risk of developing acute respiratory distress syndrome (ARDS) and to be admitted to the intensive care unit (ICU). Therefore, accurate diagnosis and monitoring of disease progression from the early stages is essential to improve the otherwise unfavourable clinical outcomes [2,8]. Higher prevalence of PE would outline the possible role of CT-PA in patients with COVID-19 infection and rapid clinical worsening [2,8] to appropriately diagnose and manage those patients.

In line with our results Lodigiani et al. reported a prevalence of 10% PE in patients undergoing CT-PA (the cumulative rate of arterial and venous thromboembolic events was of 21%) [5]. In our study this percentage does not significantly differ from the COVID-19 negative control groups during the same period in 2019 and 2020 and also not from the numbers reported in the literature [19]. This would stay in contrast to the previously believed higher incidence of thromboembolic events in COVID-19 patients [16].

However, more than 20% of our patients suspected with PE, showed signs of infarct pneumonia, despite the lack of visible filling defects in the branches of the pulmonary artery. We hypothesize, that the altered coagulation function in COVID-19 results from microscopic endothelial disruption, which leads to the formation of micro-embolism, too small to be detected on CT-PA. This is in line with a previous report from our hospital on three post-mortem case studies. Findings suggest an endothelial infection with SARS-CoV-2 leading to endotheliitis in COVID-19 of most organs including the lungs with consecutive microthrombosis [3]. This leads to the conclusion, that the only the surrogate for the underlying pathophysiologic process during COVID-19 are the secondary lung changes which become visible as peripheral wedge-shaped consolidation, as typical signs of infarct pneumonia.

Further, in more than half of our patients (regardless if with suspected PE or not) we encountered patterns normally seen in OP. This may be due to a similar histopathological mechanism: alveolar epithelial injury is accompanied by damage to the basement membrane; consecutively, plasma proteins and inflammatory cells can leak into the airspace and activate the coagulation cascade leading to fibrin deposition. Fibrin organizes into intra-alveolar fibro-inflammatory buds involving whorls of myofibroblasts in a connective tissue matrix. The final step is resorption of the inflammatory process from the centre, resulting the typical image of the reversed halo sign [9].

Additionally, in recent studies D-dimer and FDP were found to be especially predictive of disease progression and higher levels of D-dimer and FDP correlated with increased disease severity [16]; hence, patients with increased levels of D-dimers and FDP (and therefore more prone to develop thromboembolic disease) show progressive lung changes, potentially “misinterpreted” by the radiologist as an increase in infective consolidation but in reality related to microinfarction. Our hypothesis is further strengthened by the higher presence of CT signs for pulmonary artery hypertension and right heart failure in patients undergoing CT-PA or with CT patterns resembling infarct pneumonia.

Finally, we hypothesize, that the vascular pathology in COVID-19 is microscopic, and thus not *per se* visible on CT-PA due to the restricted resolution of CT. However, what we are able to see on CT, is the response of the lung as surrogate for the microangiopathic disease, showing typical changes of lung infarction and organizing pneumonia.

Different studies have shown that COVID-19 is characterized by hypercoagulability and endothelial dysfunction [2,3]. Hypercoagulability and endothelial dysfunction lead to a non-negligible number of arterial and venous thrombosis in different organ systems [21,22]. This urges the need for adequate screening procedures and antithrombotic strategies to manage thromboembolic events [23].

Our study has the following limitations: First, we do not have histopathologic confirmation of infarct pneumonia or OP of our patients. However, ethical concerns precluded the performance of lung biopsies in these patients. However preliminary histopathologic findings showed endothelial cell infection and endotheliitis which leads to impaired systemic microcirculatory function and there sequalae [3]. Further studies involving also histopathologic examination (also in form of post-mortem studies) would be needed to confirm our hypothesis. Second, the evaluated COVID-19 cohort is relatively small. Third, Dual energy (DE) CT would have been useful in the quantitative assessment of pulmonary perfusion and would have further strengthen our hypothesis of microinfarction in COVID-19 patients. However, since current guidelines advocate the use of non-contrast CT chest for the diagnosis, severity assessment, and monitoring of COVID-19 (20) we only performed non-contrast enhanced single energy CT for diagnosis and follow-up of lung consolidation in COVID-19 patients. Only if clinicians suspected PE and criteria to undergo CT-PA were present, the single energy CT-PA was performed, according to the standard procedure of our institution. However, the findings of our study would stress the need of DE-CT in COVID-19 patients to capture altered pulmonary perfusion in cases of suspected microembolic disease.

In conclusion, despite the disturbance in blood coagulation function reported in patients with COVID-19, we did not encounter higher prevalence of PE in our COVID-19 cohort compared to control the cohort 2019 or cohort 2020. However, our COVID-19 cohort showed lung changes resembling those of infarct pneumonia and OP as well as CT-signs of pulmonary-artery hypertension.

This may imply, that the vascular pathology in COVID-19 patients is of microangiopathic nature and hence generally too small to be captured directly by CT. Visible lung changes in CT might be a surrogate for the underlying pathology caused by SARS-CoV-2 unveiling the invisible endothelial changes within the lungs. An increased P/A ratio may be a hint to the underling pathology and warrant further investigation.

## Supporting information

S1 File (XLSX)
